# Cross-Cultural Adaptation of the Brazilian Portuguese Version of the Caregiver’s Feeding Styles Questionnaire

**DOI:** 10.3390/ijerph17165814

**Published:** 2020-08-11

**Authors:** Marina de Figueiredo Ferreira, Raquel de Souza Mezzavilla, Gabriela Vasconcellos de Barros Vianna, Leticia Quaresma Paolino, Haydée Serrão Lanzillotti, Ana Cristina Lindsay, Maria Helena Hasselmann

**Affiliations:** 1 Departamento de Nutrição, Universidade do Estado do Rio de Janeiro, Rio de Janeiro 20559-900, Brazil; ferreira.f.marina@gmail.com (M.d.F.F.); raquelmezzavilla@hotmail.com (R.d.S.M.); vianna.gvb@gmail.com (G.V.d.B.V.); leticia.p.quaresma@gmail.com (L.Q.P.); haydeelan@gmail.com (H.S.L.); 2 Department of Exercise and Health Sciences, University of Massachusetts Boston, Boston, MA 02906, USA; ana.Lindsay@umb.edu

**Keywords:** cross-cultural comparison, questionnaires, parenthood, food, children

## Abstract

The present study describes the cross-cultural adaptation of the Brazilian version (Rio de Janeiro) of the Caregiver’s Feeding Styles Questionnaire (CFSQ) among caregivers of children aged 3 to 6 years enrolled in a family health service in Rio de Janeiro, Brazil. The cross-cultural adaptation process included the following phases: (1) literature review; (2) translation and back-translation of the instrument; (3) assessment of semantic equivalence through cognitive interviews with caregivers; (4) discussion with experts; (5) pretesting of the revised version; and (6) assessment of psychometric characteristics, including reliability and validity of the scale. Results showed the appropriateness of the caregiver’s feeding styles concept within the Brazilian culture and that the instrument was understandable to caregivers enrolled in a family health service. The CFSQ measurements showed perfect intra-observer reliability for “demandingness” and almost perfect for “responsiveness”. Inter-observer reliability was almost perfect for both dimensions, “demandingness” and “responsiveness”. Factor analysis of the Brazilian CFSQ version proposed an instrument with one dimension and 13 items. The satisfactory results of the cross-cultural adaptation of the CFSQ suggest its applicability in the population of interest with the possible reduction of some scale items.

## 1. Introduction

Childhood overweight and obesity are public health problems worldwide, including in Brazil [1,2,3,4,5]. The family home environment plays an important role in a child’s physical, cognitive, social, and emotional development and parent–child interactions are a central part of the development of children’s lifestyle behaviors, including feeding [6]. Parents are particularly influential during the early habit-forming years. As their children’s first caregivers, disciplinarians, models, and socializing agents, parents influence the family’s home environment and children’s development of early lifestyle behaviors [6,7,8,9,10].

General parenting styles are defined as the emotional climate in the parent-child relationship, in which behaviors and attitudes are influenced by parents’ beliefs and values [11]. This definition served as a foundation for the investigation of parenting styles in the context of child feeding [11]. As proposed by Hughes et al. [12], feeding styles reflect the emotional climate in which behavioral strategies or actions are used by parents to influence children’s eating practices [12]. Therefore, the Caregiver’s Feeding Styles Questionnaire (CFSQ) developed by Hughes et al. [12] aimed to understand parents’ feeding styles. The instrument was developed specifically for low-income African American and Hispanic parents with children aged 3 to 5 years old [12].

The CFSQ consists of thirty-eight self-reported questions assessing verbal and physical feeding strategies used by parents when feeding their preschool children [12]. Building on previous research on parenting styles by Baumrind (1971) [13] and Maccoby and Martin (1983) [14], the CFSQ aimed to identify the presence of two hypothesized dimensions of child feeding, i.e., demandingness and responsiveness [12]. The instrument was developed with five response options on an intensity scale ranging from higher demandingness/responsiveness to lower demandingness/responsiveness, reflecting the following four style categories: authoritarian, authoritative, indulgent, and uninvolved [12]. Two constructs encompass these dimensions, “parent-centered feeding strategies” (with 12 items) and “child-centered feeding strategies” (with seven items). The assessment of “demandingness” is given by the average score of the 19 items (“parent-centered feeding strategies” and “child-centered feeding strategies”), and the assessment of “responsiveness” is calculated by the ratio of the mean score of the seven items (“child-centered feeding strategies” 3 + 4 + 6 + 8 + 9 + 15 + 17), subdivided by the total mean score of the 19 items (“parent-centered feeding strategies” and “child-centered feeding strategies”). Demandingness represents how much a parent encourages her/his child to eat, while responsiveness represents how parents encourage their children to eat [12].

The authoritarian feeding style embodies a high level of demandingness coupled with a low level of responsiveness, reflecting behaviors such as restricting and forcing children to eat certain foods [12]. In the authoritative style, a parent is demanding, but also dialogues and gives autonomy to their children to make their food choices [12]. Indulgent parents make some demands on what their children should eat, but they are not assertive, and usually end up allowing their children to eat what they want. In the uninvolved style, both demandingness and responsiveness are reduced, meaning children are allowed to choose what and the amount of what they eat [12].

The instrument has been widely used in the United States with ethnic minority, immigrant, and low-income families [12,15,16]. However, a limited number of studies have assessed the cross-cultural adaptation of the CFSQ [17,18,19,20], especially those that address the psychometric characteristics of the instrument. The original CFSQ shows good test-retest reliability, internal consistency, predictive, and convergent validity [12,15].

Currently, there is a lack of research examining caregivers’ child feeding styles among Brazilian parents. Developing an instrument that assesses the emotional climate at the time of feeding children would involve spending time and money on developing the dimensions and items of the instrument, and then validating the instrument to ensure that it measures what it is intended to measure. Furthermore, it would make it difficult to compare the results obtained with those found in other sociocultural contexts. Hence, cross-cultural adaptation of an instrument that has good reliability and validity is appropriate. Therefore, the present study was designed to describe and assess the phases involved in the process of cross-cultural adaptation of the Brazilian version (Rio de Janeiro) of the Caregiver’s Feeding Styles Questionnaire (CFSQ) for caregivers of children enrolled in a family health service in Rio de Janeiro (RJ), Brazil.

## 2. Materials and Methods

The cross-cultural adaptation process of the CFSQ instrument (with 19 items) was based on a universalist model developed by Herdman et al. [21], which assessed the following six types of equivalences: conceptual, of items, semantic, operational, measurement, and functional. Before the start of the study, we contacted the main author of the CFSQ (Hughes, S.) to inform her of the proposed research objectives and request permission to use the instrument. Upon receipt of permission, we proceeded to conduct the cross-cultural adaptation of the original instrument to Portuguese (Brazilian version).

### 2.1. Conceptual and Item Equivalence

The conceptual equivalence consists of exploring the construct with its explicitness and importance given to its dimensions, in the instrument of origin and in the population to which the instrument will be applied [21]. This process consists of a bibliographic review involving publications of the culture of the source instrument and the target population, followed by a discussion of the constructs with the population of interest [21]. This process is necessary to ensure that the meaning of the original construct is preserved after translation, and to explore if the different dimensions of the original instrument are present and if these are relevant and pertinent to the new context, to which the instrument is being adapted [21].

The item equivalence assesses the relevance of the indicators for capturing the dimension and is based on a bibliographic review that prioritizes publications on the processes involved in the construction of the source instrument and the bibliographic material available in the local context [21].

Therefore, in the present study, conceptual and items equivalence were assessed by carrying out the following two steps: (1) literature reviews on the instrument dimensions of “demandingness” and responsiveness” and (2) assessment of the potential use in Brazilian culture with experts in maternal and child nutrition (Rio de Janeiro).

### 2.2. Semantic Equivalence

Semantic equivalence was assessed by performing eight strategies [21]. First, the instrument was translated from English into Portuguese (Brazil), followed by back-translation in two versions, both in English. Using forward translation, the original instrument was translated from English to Portuguese by a certified translator who was native Brazilian, Portuguese speaking, and fluent in English. Next, back-translations were conducted by two professional certified native English speaker translators fluent in Portuguese. Subsequently, three Brazilian bilingual (Portuguese and English) researchers with expertise in child nutrition compared the original instrument with the translated versions to assess the following: (1) the general meaning of the items, the one that surpasses the literality of the words taking into account more subtle aspects, such as terms of the cultural context; and (2) the referential meaning of the terms/words in both cultures, the ideas to which one or several words refer. Questions related to linguist of the original version of the instrument and back-translations were reassessed by a native Brazilian, bilingual (Portuguese and English) public health researcher (ACL) with expertise in maternal and child nutrition, and parenting styles and practices in minority populations.

General meaning was assessed using a form with four response options including the following: (1) unchanged, (2) little changed, (3) much changed, or (4) completely changed. In addition, referential meaning was assessed using a scale ranging from 0% (total disagreement) to 100% (total agreement) [22,23,24].

The results of the semantic assessment suggested the need to reassess some items. Therefore, a meeting was held with a group of six experts (the first meeting) to adjust these items and to propose the preliminary version of the instrument. Using this preliminary version, cognitive interviews [25] were conducted with the population of interest comprising caregivers of children (parents, grandparents, and uncles) aged 3 to 5 years, attending the Family Health Clinic located in Rio de Janeiro, RJ (Brazil). The objectives of the cognitive interviews were the following: (1) to assess the perception of respondents about the concepts, instructions, and items and (2) to explore, with open questions, the caregivers’ understanding of the items and words suggested by the experts.

A total of 12 individual cognitive interviews were conducted with caregivers until saturation of comments about the instrument raised by child caregivers was reached [26]. The interviews were conducted by two trained interviewers, following a standardized script, which made it possible to assess if the items could be understood by the children’s caregivers; and if the instructions were clear.

Finally, a second meeting was held with the same experts to clarify questions that arose in the cognitive interviews and any unresolved questions were assessed further by a researcher with expertise in child feeding styles (ACL).

### 2.3. Operational Equivalence

Operational equivalence was performed to assess the adequacy of changes, relevance, and adequacy of the instrument, the format of the items, setting, mode of application, and categorization of responses [21]. To this end, a pretest of the final version of the adapted instrument was conducted with an additional eleven caregivers attending the same Family Health Clinic located in Rio de Janeiro, RJ (Brazil).

### 2.4. Measurement Equivalence

The psychometric characteristics, validity, and reliability of the instrument were evaluated by assessment of the measurement equivalence [21]. A confirmatory factor analysis (CFA) was performed for the dimension “demandingness/responsiveness” consisting of two constructs: “parent-centered feeding strategies” (12 items) and “child-centered feeding strategies” (7 items) using the principal factor analysis method and the likelihood ratio (LR) estimator, according to the instrument proposed by the theoretical assumptions and structural equation model [23]. This was accomplished by conducting a cross-sectional study aimed at investigating the association among family violence, parental feeding styles and practices, and overweight in children aged 2 to 9 years. The sample was comprised of 181 caregivers of children aged 3 to 6 years who attended the same Family Health Clinic.

The Kaiser–Meyer–Olkin test (KMO) verified the adequacy of the sample, using ≥0.50 as a cutoff point [27]. Dimensional scrutiny was performed by reassessing the two-dimensional structure originally proposed by Hughes et al. [12] by conducting a CFA using a structural equation model (SEM) [28]. Goodness-of-fit (GOF) was evaluated using the following three indices: (1) the root mean square error of approximation (RMSEA), which incorporates a penalty function for poor model parsimony [28] (values under 0.06 suggest close approximate (adequate) fit, whereas values above 0.10 indicate poor fit and that the model should be rejected [29]); (2) comparative fit index (CFI); and (3) Tucker–Lewis index (TLI). These represent incremental fit indices [28] contrasting the hypothesized model to a more restricted nested baseline model, the “null model”. The CFI and TLI both ranged from zero to one and values >0.9 were indicative of an adequate fit [28].

Anticipating a possible model misfit or foreseeing plausible alternative dimensional structures, the next step consisted of re-specifying the model of the CFSQ by CFA [30], using the structural equation model (SEM) procedure proposed by Reichenheim et al. [31] which proposed a one-dimensional structure and the same criteria were applied. Similarly, the fit assessment used the RMSEA, CFI, and TLI indices.

For the CFA, the generated standard matrix was examined, and standardized coefficients were analyzed according to the criterion of minimum level for the interpretation of the factor structure equal or superior to 0.30 [32]. Error variance was considered adequate when it was less than or equal to 0.70 [28].

Convergent factor validity was assessed by average variance extracted (AVE), which accounted for average variance explained by the items related to the constructs (“parent-centered feeding strategies” and “child-centered feeding strategies”), ranging from 0 to 1. AVE values ≥0.50 suggested that the items shared a high common variance [33].

Discriminant factor validity was given by comparing the square root of the AVE factor and the correlations with other factors in the system. If the square root of the AVE of a factor was greater than the correlations between it and the other factors, this discriminant validity was corroborated [28,34].

The internal consistency of each dimension used the Cronbach’s alpha point estimate and its confidence intervals (CI = 95%), and an acceptable value of 0.60 [34] was considered to be suitable. The bootstrap method with 1000 replications was used to calculate the confidence interval.

Reliability assessment was performed using the following two procedures: (1) repeatability (intra-observer reliability or test-retest, when 19 caregivers were interviewed by the same researcher at two different moments in an interval of 7 to 14 days in order to test the stability of the instrument in time and space) [20] and (2) reproducibility (inter-observer reliability, when two trained interviewers obtained responses from 48 caregivers interviewed at the same time, in order to identify possible measurement errors).

The intra- and inter-observer reliability were both estimated by the weighted Kappa coefficient of items related to the dimensions of demandingness and responsiveness and their style categories, i.e., authoritarian, authoritative, indulgent, and uninvolved [12]. Dimensions were dichotomized into high and low and the assigned values were zero and one, respectively [30]. The assessment of the weighted Kappa coefficient was based on the classification proposed by Landis and Koch [35], whose agreement is classified as poor when less than or equal to 0, slight between 0 and 0.20, fair between 0.21 and 0.40, moderate between 0.41 and 0.60, substantial between 0.61 and 0.80, and almost perfect between 0.80 and 1. All analyses were performed using the statistical software Stata version 12 [36].

### 2.5. Functional Equivalence

The assessment of functional equivalence was identified from the results of the previous stages, as a summary measure of all phases of the cross-cultural adaptation process [21,22].

### 2.6. Ethical Aspects

Participants signed the informed consent form, and the research was approved by the Research Ethics Committee of the City Hall of Rio de Janeiro (no. 1068427).

## 3. Results

### 3.1. Conceptual and Item Equivalence

The assessment of conceptual and items equivalence showed that the dimensions of the instrument (“demandingness” and “responsiveness”) can be used in Brazilian culture (Rio de Janeiro).

### 3.2. Semantic Equivalence

When analyzing semantic equivalence, the assessment of the general meaning of the items did not consider any item much or completely changed. In the assessment of the referential meaning, equivalence was higher than 80% (satisfactory) in most items, except in instructions and Item 1 (Table 1).

In Table 1 the final version refers to a more refined proposal of the CFSQ for caregivers of children (parents, grandparents, and uncles).

There were a small number of problems that were pointed out in the assessments of the general and referential meaning; however, in the meeting with the experts (first meeting), it was observed that some items would deserve attention to make the version more colloquial and improve some terms of the translation that were not adequate. In this sense, the instructions of the self-reported instrument were adapted for face-to-face interviews with changes in the verbal tense of the sentences (Table 1). Then, the modifications were tested in cognitive interviews with 12 caregivers and, in a meeting with experts (second meeting), the items that raised doubts were reassessed.

After the two meetings with the experts and the cognitive interview with the population of interest, seven items (Items 2,3,6,7,9,16, and 18) were changed to improve the translation: four (Items 1, 2, 12, and 14) due to linguistic inadequacy, three (Items 3,4, and 8) to improve the semantics, and one (Item 3) due to cultural inadequacy (Table 2).

### 3.3. Operational Equivalence

The results of this stage showed that the prototype of the Brazilian version (Rio de Janeiro) of the CFSQ (Table 1, column 7) was suitable for psychometric assessment.

### 3.4. Measurement Equivalence

Of the 181 caregivers who were interviewed, the majority (n = 153) were mothers, 82% lived in adequate housing conditions, and 53% had only one child. The KMO for the items was above 0.50 (data not presented in the table), corroborating the appropriate sample size for factor analysis. The CFA performed with the prototype of 19 items was constrained to two factors, “parent-centered feeding strategies” (12 items) and “child-centered feeding strategies” (seven items). The factor structure was designed with Factor 1 allocating items of the “parent-centered feeding strategies”, that is, Items 1, 2, 5, 7, 10, 11, 12, 13, 14, 16, 18, and 19 because they presented a standardized coefficient equal to or greater than 0.30. Among these, Items 2, 16, and 18 showed error variances above 0.70 (Table 3).

In an analogous analysis of the seven items of the “child-centered feeding strategies”, seven items were allocated in Factor 2, i.e., Items 3, 4, 6, 8, 9, 15, and 17. Of these seven items, Items 4, 6, 9, and 15 also presented a correlation standardized coefficient equal to or greater than 0.30, but with an error variance above 0.70, except for Item 5 (Table 3).

These findings made it possible to reevaluate the questionnaire by proposing a one-dimensional structure that was evaluated by structural equations model. The new prototype of the questionnaire to be tested had 13 items in a single dimension, i.e., Items 1, 2, 5, 6, 7, 10, 11, 12, 13, 14, 16, 18, and 19.

On the one hand, convergent validity was achieved in dimension Factor 1 (F1), “parent-centered feeding strategies” but not in dimension F2, “child-centered feeding strategies” (Factor 1 = 0.54 and Factor 2 = 0.32). On the other hand, discriminant validity was achieved, considering that the square roots of the AVE (Factor 1 = 0.73 and Factor 2 = 0.57) were greater than the correlations between Factors 1 and 2 (−0.11) (Table 3).

Internal consistency of the dimensions, given by Cronbach’s alpha, reached values of 0.86 (95% CI = 0.83–0.89) in Factor 1 (“parent-centered feeding strategies”) and 0.44 (95% CI = 0.31–0.57) in Factor 2 (“child-centered feeding strategies”). From the findings, it can be concluded that Factor 1 showed expressive internal consistency, even when considering the lower limit of the confidence interval. However, Factor 2 was not able to achieve desirable internal consistency. This is another evidence for proposing the one-dimensional questionnaire.

The goodness-of-fit (GOF) presented the following results: RMSEA = 0.07, CFI = 0.85, and TLI = 0.83, which indicated that is still not quite adequate. This showed the need to re-specify the model to a one-dimensional structure (presented below in Table 4). The intra-observer reliability, test-retest (n = 19), revealed perfect agreement for the dimension of “demandingness” (1.00), and almost perfect for “responsiveness” (0.87, 95% CI 0.63–1.00). Inter-observer reliability (n = 48) was almost perfect for the dimensions of “demandingness” (0.95, 95% CI 0.86–1.00) and “responsiveness” (0.95, 95% CI 0.86–1.00) (Table 3). In the re-specified model, Table 4, the structure of the questionnaire presents values greater than 0.30, but five items (2, 6, 14, 16, and 18) presented an error variance greater than 0.70. Convergent validity is achieved (AVE = 0.57) and there is no reason to evaluate discriminate validity since it is a one-dimensional structure. The internal consistency of the questionnaire was achieved with reasonably expressive Cronbach’s alpha values, considering the point estimate and its confidence interval. The model’s fit was adequate (RMSEA <0.10, CFI = 0.90, TLI = 0.89, Table 4). These findings allow us to infer that the CFSQ, when subjected to the process of cross-cultural adaptation for the Brazilian language (Rio de Janeiro), can have a one-dimensional structure.

### 3.5. Functional Equivalence

Evidence provided by the other equivalences allows us to infer the range of functional equivalence.

## 4. Discussion

The present study describes the cross-cultural adaptation of the Brazilian version (Rio de Janeiro) of the Caregiver’s Feeding Styles Questionnaire (CFSQ) [12]. To our knowledge, this is the first study to validate a version of the CFSQ specifically modified for use with Brazilian mothers and caregivers of young children enrolled in a Family Health Service (aka “*Programa Saúde da Família*”). Some modifications were made to adjust linguistic and cultural inadequacies and keep the original meaning of the instrument in English.

Regarding the stage of semantic equivalence, although at the first meeting with the experts they suggested semantic changes in terms and some items, such as “your son” for “your child”, “meal” for “dinner”, and “physically struggles with the child” for “physically forces the child”. At the second meeting, after the cognitive interviews, we chose to keep its original translation for the following reasons: keep “your child”, because the instrument is up to other caregivers and not just fathers and mothers; keep “dinner”, because, for the population of interest, “dinner” is a family meal, thus, is not inadequate; keep “physically struggles with the child”, because the results of the cognitive interviews showed that the translated expression, as well as the offered example, were understood as having the same connotation of the original instrument “the child’s self-control”. It is worth mentioning that in an adaptation study with low-income African American and Hispanic parents [16] this item was changed, aiming at a better understanding of the question, and another example was included in the item. However, this modification was not considered to be necessary in the Brazilian version.

It is also worthy of comment that the example of Item 14 “will not get fruit” aims to capture the control/restriction of foods of interest to the child. In the meeting with the experts, this example was discussed because they believed that, in the target population, not giving the fruit to the child would not be a form of control in many cases. Hughes et al. [16], in another adaptation study, have replaced this example by ”will not get ice cream”. However, the target population identified fruit as one of the children’s favorite foods, therefore, it was decided to keep the original example.

In the measurement stage, using the questionnaire in its two-dimensional form with 19 items, the correlations were very low for Items 3 (0.23), 8 (0.08), and 17 (0.28), items that are part of Factor 2 “child-centered feeding strategies” (Table 3). Analyzing the semantic evaluation among the three evaluators, values of 90% are verified for Items 3 and 8, and 100% for Item 17 (Table 1), which are considered to be adequate, requiring small semantic adjustments for Items 3 and 8 (Table 2). The same items, in the original questionnaire [12], presented factorial loads of 0.57, 0.57, and 0.43, respectively. Analyzing its contents, Item 3 “encourage the child to eat by arranging the food to make it more interesting (for example, making smiley faces on the pancakes)”, Item 8 “allow the child to choose the foods he or she wants to eat for dinner from foods already prepared”, and Item 17 “help the child to eat dinner (for example, cutting the food into smaller pieces)”, it appears that the problem with the items is more cultural than semantic, since these practices (especially Items 3 and 8), centered on the child, are not part of the daily life of the target population, which points to the need for re-specification of the model.

Item 3 “encourage the child to eat by arranging the food to make it more interesting (for example, making smiley faces on the pancakes)” was rewritten because of cultural inadequacy. To arrange the food “in a more interesting way”, is not a practice adopted by the vast majority of Brazilian parents, which explains the item’s inadequacy in the target population. In an adaptation study for Portuguese (Porto Alegre, RS), the authors also proposed changes in Item 3 (“encourages the child to eat, arranging the food to make it more interesting (for example, making figures/designs with vegetables”), which suggests that this item for Brazilian culture should be used carefully [20].

Concerning Item 8 “allow the child to choose the foods he or she wants to eat for dinner from foods already prepared”, the requirement was only to improve the semantic adequacy (Table 2). Unlike the original instrument, Item 8, which was allocated to the “child-centered” factor, migrated to the “parent-centered” factor in this study. The possible interpretation for this finding in Brazilian culture is that the choice of food is a parental decision, impairing the child’s autonomy. We consider that these obstacles are of semantic origin, and also characteristics of the Brazilian culture, assuming that this item is not a strategy that parents use to promote the child’s autonomy.

Regarding Item 17 “help the child to eat dinner (for example, cutting the food into smaller pieces”, which in the original instrument was also allocated to the “child-centered feeding strategies” factor, in the present study, also moved to the “parent-centered feeding strategies” factor. Once again, this reflects possible parental control in relation to the food intake chosen by them, consequently, interfering in the child’s autonomy. Evidence suggests that parental control strategies to regulate food consumption can decrease the ability of children to self-regulate their appetite, increasing the risk of childhood obesity [6,37]. Nonetheless, a previous study conducted with low-income Latin American mothers living in the USA showed that mothers participating in the study held positive beliefs about the use of food to shape children’s behavior [37]. This finding indicates the importance of addressing cultural influences on parents’ behaviors that influences a child’s self-regulation that ultimately influences a child’s weight status.

Marked discrepancy between the factor load of Item 7 “say something to show your disapproval of the child for not eating dinner” (Factor 1, “parent-centered”) was found as compared with the original by Hughes et al. [12]. In the original study, the factor load was 0.37, but in the present study, it reached 0.71, although, among the evaluators, the semantic agreement reached 87%. Similarly, Item 8 “allow the child to choose the foods he or she wants to eat for dinner from foods already prepared” (Factor 2, “child-centered”) presented factorial load at the cutoff threshold (0.30) in the original study [12], whereas, in the present study, it was extremely low (0.08), even though the semantic agreement between the evaluators reached 90%. Once again, it can be inferred that, in this target population, the biggest issue is not semantics, but a culture in which parents must exercise control over child feeding. They disapprove of the child for not eating and do not allow the child to choose the foods he/she wants to eat. For reasons that go beyond the present study, such as economic and educational issues, the results indicate a predominance of parental/caregiver control over their children, which represents more evidence for the re-specification of the model.

Contrary to the results reported by Hughes et al. [12], which proposed the two-dimensional questionnaire, our results pointed to a one-dimensional structure with the exclusion of the items that presented low coefficients. Therefore, the model was re-specified.

In the re-specified model (Table 4), the coefficients presented themselves above 0.40, except for Item 16, which was close to the cutoff point (0.33). For this item, a very expressive change was needed (Table 1), but even with that, the item was not quite adequate for capturing the latency of parental feeding strategies.

It is important to emphasize that Item 6 “reason with the child to get him or her to eat (for example, “milk is good for your health because it will make you strong”)”, which in the two-dimensional model (Table 3) presented a coefficient of 0.59, the best among the others in Factor 2 “child-centered feeding strategies”, had its coefficient reduced to 0.47 in the one-dimensional model (Table 4). This strategy represents a way of making a child reflect and encourages the child to eat. But, when “centered on parents”, it can be interpreted as an external pressure to eat. This seems to suggest that parents use a diversity of strategies directed to food, and they are both positive and imperative. According to Costanzo and Woody [38], the same parent had different styles depending on the moment of the child’s development and did not present a single style. Despite the restructuring of the model, some error variances remained high (Items 2, 6, 14, 16, and 18).

In the present study, the two-dimensional model (Table 3) showed Cronbach’s alpha of 0.86 and 0.45 for Factor 1 (parent-centered) and Factor 2 (child-centered), respectively. In the one-dimensional model (re-specified) the value reached 0.87 (Table 4). Confronting Cronbach’s alpha from the original questionnaire [12] (two-dimensional), with values of 0.86 and 0.71 for Factors 1 and 2, respectively, it appears that the one-dimensional (re-specified) model of the present study showed improvement on the questionnaire’s internal consistency.

With regard to validity, the original instrument [12] assessed the convergent validity of feeding styles by comparing them with two instruments (Child Feeding Questionnaire and Parenting Dimensions Inventory-S) through general multivariate linear modeling, using ANOVA to assess the mean differences in parenthood and the existing measures of authoritarian feeding practices in the four feeding styles. In this study, we chose the convergent and discriminant factor validity, a methodological option that did not allow us to make comparisons between findings.

The adjustment evaluation of the two-dimensional model (Table 3), given by RMSEA, CFI, and TLI indices, showed values of 0.07, 0.85, and 0.83, respectively. In contrast, in the one-dimensional model (Table 4), the indices revealed different values, i.e., 0.07, 0.90, and 0.89, respectively, showing a better fit of the one-dimensional model.

Hughes et al. [12] established the reliability of the original instrument by Pearson correlation, using the sum of the scores of the two dimensions “demandingness” (parent-centered feeding strategies and child-centered feeding strategies) and “responsiveness” (child-centered feeding strategies), in one test-retest. The correlation values were 0.85 and 0.82 for each dimension. However, the study did not assess intra- and inter-respondent agreement. In the present study, the dimensions “demandingness” and “responsiveness” were assessed by scores on a dichotomous scale, and this form of analysis made it possible to measure the intra- and inter-respondent by weighted Kappa, which showed reliability values higher than those found by Hughes et al. [12] (Table 3).

The cross-cultural adaptation of the CFSQ to the Brazilian Portuguese language can be considered to be an advancement for measuring the behavior of parents when feeding their children. It is important to highlight that this study was intended to translate the CFSQ for caregivers of children enrolled in a Family Health Program (aka “*Programa Saúde da Família”* or PSF). When changing the target population, the questionnaire should be reevaluated.

## 5. Conclusions

The relevance of this study is in the use of the recommended procedures for cross-cultural adaptations proposed by Herdman et al. [21], in addition to using more than two versions of the back-translation of the instrument. This performance made it possible to better understand the performed translation, use techniques of cognitive interviews to further refine the version of the instrument with the target population, and perform the pretest stage of the instrument to detect possible flaws in the version and also reveal psychometric properties of the scale. It would be interesting to use the psychometric study in other contexts (including Brazilian) to observe the behavior of factor loadings [22], as well as a further investigation with qualitative studies.

The results of the cross-cultural adaptation of the Brazilian version (Rio de Janeiro) of the CFSQ suggest its applicability in the population of interest with the possible reduction of some items (Items 3, 4, 8, 9, 15, and 17), particularly from dimension two, in the scale. Since infant overweight is a major public health problem with negative consequences for child and adult health, it is relevant to encourage future research in Brazil and other countries with Brazilian immigrants.

In the current epidemiological scenario, we believe that it is crucial to investigate parental feeding styles as a determinant of the relationship between feeding practices in childhood and the etiology of obesity. Understanding psychobiological aspects of child eating behavior can help the development of programs for the prevention and treatment of child obesity.

## Figures and Tables

**Table 1:** Original version, translation, back-translations A and B, Synthesis of General and Referential Semantic Assessment, and the Brazilian final version (Rio de Janeiro) of the Caregiver’s Feeding Styles Questionnaire (CFSQ), Family Health Clinic, Rio de Janeiro, Brazil, 2015–2017.

Original	Translation	Back-	Back-	Synthesis		Final Version
		Translation A	Translation B	Eval. 1, 2 e 3		
				G	R	
Caregiver’s Feeding Styles Questionnaire	Questionário dos Estilos de Alimentação dos Cuidadores	Caregiver’s Feeding Style Questionnaire	Caregiver’s Feeding Styles Questionnaire	U	100%	Questionário dos Estilos de Alimentação dos Cuidadores
These questions deal with your interactions with your preschool child during the dinner meal. Circle the best answer that describes how often these things happen. If you are not certain, make your best guess. How often during the dinner meal do you ….	Estas perguntas abordam a sua interação com sua criança em idade pré-escolar durante o jantar. Circule a melhor resposta que descreve com que frequência estas coisas acontecem. Se você não tem certeza dê a sua melhor estimativa Quantas vezes durante a refeição do jantar você …	The questions below address your interaction with your preschool-aged child during the evening meal. Circle the best answer, which describes the frequency with which these things occur, if you are unsure, give your best estimate. How many times during the evening meal do you…	These questions approach your interaction with your preschool child during dinner. Circle the answer that best describes how often these things happen If you’re not sure, give your best estimate. How often during the dinner meal do you…	SC	77%	Estas perguntas abordam como você lida com a sua criança durante o Jantar. Escolha a melhor resposta [mostrar cartão] que descreve com que frequência estas coisas acontecem. Se você não tem certeza, diga a que melhor se aproxima. Com que frequência durante o Jantar você…
Reply Options: Never/Rarely Sometimes/Most of the time/Always	Opções de respostas: Nunca/Raramente/Ás vezes/Maioria do tempo/Sempre	Reply Options: Never/Rarely/At times/ Most of the time/ Always	Reply Options: Never/Rarely/Sometimes/Most of the time/Always	U	100%	Opções de respostas: Nunca/Raramente /Ás vezes/Maioria do tempo/Sempre
1.Physically struggle with the child to get him or her to eat (for example, physically putting the child in the chair so he or she will eat).	1. Se esforçar fisicamente com a criança para fazer com que ela coma (por exemplo, fisicamente colocando a criança na cadeira para que coma).	1. Use physical force with the child to ensure that he/she eats (for example, physically placing the child in a chair in order to make him/her eat).	1. Physical try to make your child eat (for example, physically putting the child in the chair to eat).	SC	78%	1. Se esforça fisicamente com a criança para fazer com que ela coma (por exemplo, forçá-la a ficar na cadeira para que coma).
2.Promise the child something other than food if he or she eats (for example, “If you eat your beans, we can play ball after dinner”).	2. Prometer algo além da comida para a criança se ele comer (por exemplo, “se você comer o feijão, você pode jogar bola depois do jantar”).	2. Promise something in addition to food to the child if he/she eats (for example, “If you eat the beans, you can play football after supper”).	2. Promise something other than food so the child will eat (for example, “if you eat your beans, you can play ball after dinner”).	U	92%	2. Promete algo diferente da comida para a criança se ela comer (por exemplo, “se você comer o feijão, nós podemos jogar bola depois do jantar”).
3. Encourage the child to eat by arranging the food to make it more interesting (for example, making smiley faces on the pancakes).	3. Motivar a criança para comer arrumando os alimentos de uma maneira mais interessante (por exemplo, fazendo rostos sorridentes nas panquecas).	3. Motivate the child to eat by arranging the food in a more interesting manner (for example, making smiling faces on the pancakes).	3. Encourage the child to eat by arranging the food in a more interesting way (for example, making smiling faces on pancakes).	SC	90%	3. Estimula a criança para comer, arrumando os alimentos de uma maneira mais interessante (por exemplo, fazendo rostos sorridentes na comida ou qualquer coisa que deixe o prato mais bonito).
4. Ask the child questions about the food during dinner.	4. Fazer perguntas para criança sobre a comida durante o jantar.	4. Ask the child questions about the food during supper	4. Ask the child questions about the food during dinner.	U	100%	4. Faz perguntas para a criança sobre a comida que ela está comendo.
5. Tell the child to eat at least a little bit of food on his or her plate.	5. Dizer para a criança comer pelo menos um pouco da comida que está no seu prato.	5. Tell the child to eat at least some of the food that is on his/her plate.	5. Tell the child to eat at least a bit of the food on his or her plate.	U	100%	5. Diz para a criança comer pelo menos um pouco da comida que está no seu prato.
6. Reason with the child to get him or her to eat (for example, “Milk is good for your health because it will make you strong”).	6. Tentar raciocinar com a criança para fazer com que coma (por exemplo, “Leite é bom para a sua saúde pois te fará ficar forte”).	6. Try to reason with the child to make him/her eat (for example, “Milk is good for your health as it will make you strong”).	6. Try to reason with the child so that he or she will eat (for example, “Milk is good for your health because it will make you stronger”).	SC	95%	6. Argumenta com a criança para fazer com que coma (por exemplo, “Leite é bom para a sua saúde pois fará você ficar forte”).
7. Say something to show your disapproval of the child for not eating dinner.	7. Dizer alguma coisa para mostrar a sua decepção por não comer o jantar.	7. Say something to show that you are disappointed that he/she has not eaten supper.	7. Say something to show your disappointment that the child is not eating dinner.	U	87%	7. Diz alguma coisa para mostrar a sua desaprovação por ela não comer o jantar
8. Allow the child to choose the foods he or she wants to eat for dinner from foods already prepared.	8. Permitir que a criança escolha os alimentos que quer comer no jantar dos alimentos já preparados.	8. Allow the child to select the food that he/she wants to eat for supper from among the food already prepared.	8. Let the child choose the foods he or she wants to eat for dinner among the foods prepared.	SC	90%	8. Permite que a criança escolha os alimentos que quer comer entre os alimentos já preparados.
9. Compliment the child for eating food (for example, “What a good boy! You’re eating your beans”).	9. Elogiar a criança por comer os alimentos (por exemplo, “Que menino legal! Você está comendo seu feijão”).	9. Praise the child for eating the food (for example, “What a good boy/girl! You are eating your beans”).	9. Praise the child for eating the food (for example, “What a good boy! You’re eating your beans”).	U	100%	9. Elogia a criança por comer algum alimento (por exemplo, “Que menino legal! Você está comendo seu feijão”).
10. Suggest to the child that he or she eats dinner, for example by saying, “Your dinner is getting cold”.	10. Sugerir que a criança coma seu jantar, por exemplo, ao dizer, “Seu jantar está esfriando”.	10. Suggest that the child eats his/her supper by saying, for example, “Your supper is getting cold”.	10. Suggest that the child eat dinner, by saying, “Your dinner is getting cold.”	U	100%	10. Sugere que a criança coma a comida (por exemplo, ao dizer, “Sua comida está esfriando”).
11. Say to the child “Hurry up and eat your food”.	11. Dizer para a criança “Vamos logo e coma sua comida”.	11. Say to the child “Come on, eat your food”.	11. Tell the child, “Go ahead and eat your dinner.”	U	100%	11. Diz para a criança “Vamos logo e coma sua comida”.
12. Warn the child that you will take away something other than food if he or she doesn’t eat (for example, “If you don’t finish your meat, there will be no play time after dinner”).	12. Avisar a criança que você vai tirar alguma coisa dela que não é comida se ela não comer (por exemplo, “Se você não terminar de comer a sua carne, não haverá tempo para brincar depois do jantar”).	12. Inform the child that you will deprive him/her of something that is not food if he/she fails to eat (for example, “If you don’t eat all your meat, you will not have time to play after supper”).	12. Tell the child that you’re going to take something away other than food if he or she doesn’t eat (for example, “If you don’t finish eating your meat, there won’t be time to play after dinner”).	U	95%	12. Avisa a criança que você vai tirar alguma coisa dela (que não é comida) se ela não comer (por exemplo, “Se você não terminar de comer a sua carne, não vai brincar depois do jantar”).
13. Tell the child to eat something on the plate (for example, “Eat your beans”).	13. Dizer para a criança para comer alguma coisa que está no prato (por exemplo, “Coma seu feijão”).	13. Tell the child to eat something that is on the plate (for example, “Eat your beans”).	13. Tell the child to eat something on the plate (for example, “Eat your beans”.	U	100%	13. Diz para a criança comer alguma coisa que está no prato (por exemplo, “Coma seu feijão”).
14. Warn the child that you will take a food away if the child doesn’t eat (for example, “If you don’t finish your vegetables, you won’t get fruit”).	14. Avisar a criança que você vai tirar alguma comida se a criança não comer (por exemplo, “se você não terminar seus legumes, você não vai ganhar fruta”).	14. Inform the child that you will deprive him/her of some food if he/she does not eat (for example, “If you don’t finish your vegetables, you are not having any fruit”).	14. Tell the child you’re going to take some food away if he or she doesn’t eat (for example, “If you don’t finish your vegetables, you won’t get any fruit”).	U	92%	14. Avisa para a criança que você vai tirar algum alimento que ela goste se ela não comer (por exemplo, “se você não terminar seus legumes, você não vai ganhar fruta”).
15. Say something positive about the food the child is eating during dinner.	15. Dizer alguma coisa positiva sobre a comida que a criança está comendo no jantar.	15. Say something positive about the food that the child is eating for supper	15. Say something positive about the food the child is eating for dinner.	U	100%	15. Diz alguma coisa positiva sobre a comida que a criança está comendo.
16. Spoon-feed the child to get him or her to eat dinner.	16. Dar comida na boca com colher para fazer com que a criança coma o seu jantar.	16. Feed the child with a spoon to make him/her eat him/her supper.	16. Spoon-feed the child to make him or her eat dinner.	U	90%	16. Dá comida na boca para fazer com que a criança coma.
17. Help the child to eat dinner (for example, cutting the food into smaller pieces).	17. Ajudar a criança a comer o jantar (por exemplo, cortando os alimentos em pedaços menores).	17. Help the child to eat his/her supper (for example, by cutting up the food into smaller places).	17. Help the child eat dinner (for example, cutting the food into smaller pieces).	U	100%	17. Ajuda a criança a comer (por exemplo, cortando os alimentos em pedaços menores).
18. Encourage the child to eat something by using food as a reward (for example, “If you finish your vegetables, you will get some fruit”).	18. Motivar a criança a comer alguma coisa ao usar alimentos como recompensa (por exemplo, “Se você comer todos os legumes, você ganha algumas frutas”).	18. Motivate the child to eat some food by using food as a reward (for example, “If you eat all your vegetables, you will get some fruit”).	18. Encourage the child to eat something by using foods as a reward (for example, “If you eat all your vegetables, you get some fruit”).	U	92%	18. Estimula a criança a comer a comida usando alimentos como recompensa (por exemplo, “Se você comer todos os legumes, você ganha algumas frutas”).
19. Beg the child to eat dinner.	19. Implorar para a criança comer seu jantar.	19. Plead with the child to eat his/her	19. Beg the child to eat dinner.	U	100%	19. Implora para a criança comer sua comida.

CFSQ, Caregiver’s Feeding Styles Questionnaire; Eval, evaluators; G, general meaning; R, referential meaning; U, unchanged; SC, slightly changed.

**Table 2:** Items translated from the original, changes, and justifications for the Brazilian final version (Rio de Janeiro) of the CFSQ, Family Health Clinic, Rio de Janeiro, Brazil, 2015–2017.

Items Translated	Changes	Justifications
1. Se esforçar fisicamente com a criança para fazer com que ela coma (por exemplo, fisicamente colocando a criança na cadeira para que coma).	“fisicamente colocando a criança na cadeira” por “forçá-lo a ficar na cadeira”	Linguistic inadequacy
2. Prometer algo além da comida para a criança se ela comer (por exemplo, “se você comer o feijão, você pode jogar bola depois do jantar”).	1st “algo além da comida” por “algo diferente de comida”	Linguistic inadequacy
	2nd “você pode jogar bola” por “nós podemos jogar bola”	Keep the original meaning in English
3. Motivar a criança para comer arrumando os alimentos de uma maneira mais interessante (por exemplo, fazendo rostos sorridentes nas panquecas).	1st “motivar” por “estimula”.	Keep the original meaning in English
	2nd “panquecas” por “comida”	Cultural inadequacy
	3rd incluir o exemplo, “ou qualquer coisa que deixe o prato mais bonito”.	Improve the semantics
4. Fazer perguntas para criança sobre a comida durante o jantar.	“comida durante o jantar” por “comida que ele está comendo”.	Improve the semantics
6. Tentar raciocinar com a criança para fazer com que coma (por exemplo, “Leite é bom para a sua saúde pois te fará ficar forte”).	“tentar raciocinar” por “argumenta com”.	Keep the original meaning in English
7. Dizer alguma coisa para mostrar a sua decepção por não comer o jantar.	“decepção” por “desaprovação”.	Keep the original meaning in English
8. Permitir que a criança escolha os alimentos que quer comer no jantar dos alimentos já preparados.	“comer no jantar os alimentos já preparados” por “comer entre os alimentos já preparados”.	Improve the semantics
9. Elogiar a criança por comer os alimentos (por exemplo, “Que menino legal! Você está comendo seu feijão”).	“comer os alimentos” por “comer algum alimento”.	Keep the original meaning
12. Avisar a criança que você vai tirar alguma coisa dela que não é comida se ela não comer (por exemplo, “Se você não terminar de comer a sua carne, não haverá tempo para brincar depois do jantar”).	“não haverá tempo para brincar” por “não vai brincar”.	Linguistic inadequacy
14. Avisar a criança que você vai tirar alguma comida se a criança não comer (por exemplo, “se você não terminar seus legumes, você não vai ganhar fruta”).	“alguma comida se a criança não comer” por “algum alimento que ele goste se ele não comer”.	Linguistic inadequacy
16. Dar comida na boca com colher para fazer com que a criança coma o seu jantar.	retira as palavras “com colher”	Keep the original meaning in English
18. Motivar a criança a comer alguma coisa ao usar alimentos como recompensa (por exemplo, “Se você comer todos os legumes, você ganha algumas frutas”).	“motivar” por “estimula”.	Keep the original meaning in English

CFSQ, Caregiver’s Feeding Styles Questionnaire.

**Table 3:** Standardized coefficient of the 19 items (Factors 1 and 2) by structural equation model, error variance, Cronbach’s alpha, and reliability by weighted Kappa of the Brazilian (Rio de Janeiro) of the two-dimensional prototype of the CFSQ, Rio de Janeiro, Brazil, 2015–2017.

Items	Standardized Coefficient		Error Variance
	Feeding Strategies		
	F1: Parent-Centered	F2: Child-Centered	
1	0.56	*	0.68
2	0.46	*	0.78 *
3	*	0.23	0.95
4	*	0.34	0.88
5	0.70	*	0.51
6	*	0.59	0.65
7	0.71	*	0.50
8	*	0.08	0.99
9	*	0.33	0.89
10	0.62	*	0.61
11	0.69	*	0.53
12	0.57	*	0.67
13	0.76	*	0.42
14	0.54	*	0.70
15	*	0.39	0.84
16	0.33	*	0.89
17	*	0.28	0.92
18	0.43	*	0.81
19	0.64	*	0.58
AVE	0.54	0.32	
AVE root	0.73	0.57	
Factor oblimin rotation matrix (Correlação F1 versus F2)			
	F2: Child-centered		
F1: Parent-centered	−0.11		
Cronbach’s alpha			
	F1: Parent-centered	F2: Child-centered	
	0.86	0.44	
	(95% CI 0.83–0.89)	(95% CI 0.31–0.57)	
Goodness of Fit			
	RMSEA	0.07	
		95% CI 0.05–0.08	
	CFI	0.85	
	TLI	0.83	
Reliability			
Dimension	Intra-observer Repeatability (weighted Kappa) (n = 19)	Inter-observer Reproducibility (weighted Kappa) (n = 48)	
**Demandingness**	1.00	0.95 (95% CI 0.86–1.00)	
**Responsiveness**	0.87 (95% CI 0.63–1.00)	0.95 (95% CI 0.86–1.00)	

F1, Factor 1; F2, Factor 2; AVE, average variance extracted; 95% CI, 95% confidence interval. (*) The item does not belong to the factor (F1: 3, 4, 6, 8, 9, 15, 17 and F2: 1, 2, 5, 7, 10, 11, 12, 13, 14, 16, 18, 19); AVE, average variance extracted; RMSEA, root mean square error of approximation; CFI, comparative fit index; TLI, Tucker–Lewis index.

**Table 4:** Standardized coefficient of the 13 items (one-dimensional structure) by structural equation model, error variance, and Cronbach’s alpha of the Brazilian (Rio de Janeiro) final version of the CFSQ, Rio de Janeiro, Brazil, 2015–2017.

Items	Standardized Coefficient	Error Variance
	Feeding Strategies	
1	0.57	0.68
2	0.47	0.78
5	0.69	0.52
6	0.47	0.78
7	0.71	0.50
10	0.62	0.62
11	0.69	0.52
12	0.57	0.67
13	0.75	0.43
14	0.54	0.71
16	0.33	0.89
18	0.43	0.81
19	0.65	0.57
AVE	0.57	
Cronbach’s alpha		
0.87		
(95% CI 0.84–0.89)		
	Goodness of Fit	
RMSEA	0.07	
	95% CI 0.05–0.09	
CFI	0.90	
TLI	0.89	

AVE, average variance extracted; 95% CI, 95% confidence interval; RMSEA, root mean square error of approximation; CFI, comparative fit index; TLI, Tucker–Lewis index.
